# Real-world data mining meets clinical practice: Research challenges and perspective

**DOI:** 10.3389/fdata.2022.1021621

**Published:** 2022-10-21

**Authors:** Federica Mandreoli, Davide Ferrari, Veronica Guidetti, Federico Motta, Paolo Missier

**Affiliations:** ^1^Department of Physics, Informatics and Mathematics, Università di Modena e Reggio Emilia, Modena, Italy; ^2^Department of Population Health Sciences, King's College London, London, United Kingdom; ^3^Guy's and St. Thomas' NHS Fundation Trust, London, United Kingdom; ^4^Deutsches Elektronen–Synchrotron DESY, Hamburg, Germany; ^5^School of Computing, Newcastle University, Newcastle upon Tyne, United Kingdom

**Keywords:** artificial intelligence, electronic health records, high-stakes domains, machine learning, P4 medicine

## Abstract

As Big Data Analysis meets healthcare applications, domain-specific challenges and opportunities materialize in all aspects of data science. Advanced statistical methods and Artificial Intelligence (AI) on Electronic Health Records (EHRs) are used both for knowledge discovery purposes and clinical decision support. Such techniques enable the emerging Predictive, Preventative, Personalized, and Participatory Medicine (P4M) paradigm. Working with the Infectious Disease Clinic of the University Hospital of Modena, Italy, we have developed a range of Data–Driven (DD) approaches to solve critical clinical applications using statistics, Machine Learning (ML) and Big Data Analytics on real-world EHR. Here, we describe our perspective on the challenges we encountered. Some are connected to medical data and their sparse, scarce, and unbalanced nature. Others are bound to the application environment, as medical AI tools can affect people's health and life. For each of these problems, we report some available techniques to tackle them, present examples drawn from our experience, and propose which approaches, in our opinion, could lead to successful real-world, end-to-end implementations.

**DESY report number:**

DESY-22-153.

## 1. Introduction

Real-world Data Mining (DM) is characterized by the application of ML and DM techniques to datasets that exist in-the-wild. Specifically, research based on retrospective studies start from datasets that are not *research ready*, and thus require substantial engineering alongside a deep understanding of the data domain.

Underpinned by Big Health Data, including EHRs, but also Patient Reported Outcomes (PROs) like mobile-based questionnaires and wearable devices, DM and AI can significantly advance the P4M vision focused on improving people's wellness by personalizing care (Flores et al., 2013). Drawing from our collaboration with the Infectious Disease Clinic of the University Hospital of Modena, Italy, in this paper we discuss principled solutions to demanding challenges in P4M, and present our perspectives on open research problems.

A useful initial distinction should be made between prospective and retrospective datasets. The former tend to be collected for research purposes, and are thus stable, being the result of an agreed-upon protocol. However, they are expensive to generate and often present limited opportunities to answer different research questions (reusability). In contrast, retrospective datasets such as EHRs, containing lifetime patients' histories, include a wide variety of observations. In this paper, we primarily focus on the challenges associated with making such datasets research ready. Moreover, following the dominant approach in predicting clinical outcomes (Kuan et al., 2021), we consider applying supervised learning algorithms to these datasets. Such algorithms impose strict requirements on the training set in terms of overall volume, completeness, balance among target classes, consistency, and regularity of observations over time. We refer to the lack of these requirements as an *intrinsic* data issue as these properties must be addressed before training ML models. These problems, taken separately, are all well-known in the literature, and examples and solutions abound for each of them. Our suggestion is that the real challenge is *their interplay*. Indeed, health datasets are often **scarce**, i.e., lack a substantial fraction of the data, often according to recognizable patterns. They also tend be **imbalanced**, as some classes are typically under-represented. They often show **time inconsistencies** due to changes in data collection protocols and the heterogeneity in the data sources. Finally, patients' medical histories are naturally **irregular** over time.

Other challenges exist at the interface between ML and medicine, resulting in further human-in-the-loop requirements. In medicine, not all errors are qualitatively the same. Using different models or the predisposition toward specific error types leads to different clinical decisions. Furthermore, only few human-in-the-loop systems exist that accept knowledge-based feedback from physicians. We refer to these additional challenges as *translational*.

Intrinsic and translational challenges are discussed in Sections 2 and 3, respectively, and exemplified by leveraging examples coming from the following studies:

- My Smart Age with HIV (MySAwH) (Ferrari et al., 2020a): an international multi-center prospective study aimed at studying and monitoring healthy aging in People Living with HIV (PLWH). Collected data came from routine clinical assessments and innovative PROs, collected through mobile and wearable devices;
- Ferrari et al. (2020b,c) and Mandreoli et al. (2021): retrospective studies focused on the hospital resource management and clinical decision making problems emerged during the Covid–19 pandemic.

Following Jung (2022), in Table 1 we summarize the data points, features, outcomes, and ML techniques used in the aforementioned studies.

**Table 1:** Details about the ML applications, brought as examples.

**Study**	**Ferrari et al. (2020a)**	**Ferrari et al. (2020b,c)**	**Mandreoli et al. (2021)**
Data points	4,176 EHRs 261 patients	1, 068 EHRs 198 patients	14, 249 EHRs 1,040 patients
Features	Total = 96 56) PROs 27) Blood tests 7) HIV specific 3) Body composition 3) Activity tracking	Total = 91 39) Blood and urine tests 29) Symptoms 16) Comorbidities 7) BGA	Total = 17 10) Blood and urine tests 3) Respiratory specific 2) BGA, oxygen–therapy 2) Age, CCI
Outcome(s)	Falls, SPPB, QoL	Respiratory failure	Oxygen–therapy state
Domain	∈ℝ	∈{0, 1}	∈{*s* ≤ *N*∣*s*∈ℕ^+^}
ML model	GBM/DT Regressor(s)	GBM/DT Classifier	HMM–ensemble
Explainability	SHAPs	SHAPs	✘
Loss function(s)	MAPE	Binary Cross–entropy	*E*_β = 0.5_ *effectiveness* measure
		(tuned to minimize FN rate)	as in van Rijsbergen (1979)
Challenges			
- Sparsity	✓	✓	✓
- Scarcity	✓	✓	✓
- Imbalance	✘	✓	✓
- Instability	✘	✓	✓
- Error preference	✘	✓	✓
- Human in-the-loop	✓	✓	✘

BGA, Blood Gas Analysis; CCI, Charlson Comorbidity Index; EHRs, Electronic Health Records; FN, False Negative; GBM/DT, Gradient Boosting Machine/Decision Tree; HIV, Human Immunodeficiency Virus; HMM, Hidden Markov Model; MAPE, Mean Average Percentage Error; ML, Machine Learning; PROs, Patient Reported Outcomes; QoL, Quality of Life; SHAPs, SHapley Additive exPlanations values; SPPB, Short Physical Performance Battery.

## 2. Intrinsic data challenges

The interplay of data scarcity, sparsity, target unbalancing, and instability issues is frequent in both prospective and retrospective health datasets. In what follows we briefly summarize this interaction and describe present and future techniques to manage it.

### 2.1. Sparsity/scarcity and (im)balance

Data sparsity refers to missing values within records or time series. Scarcity, in contrast, refers to insufficient number of observations, e.g., the number of patients' records in classification tasks.

The primary source of retrospective studies, operational EHRs that are collected in-the-wild, are time series of patient events (primary/secondary care, prescriptions), and can be irregular (events are collected only when they happen), scarce (healthy individuals will have fewer events), sparse and heterogeneous (the variables represented within each event, for instance clinical tests, vary depending on the condition). However, a distinction between non-events, which are informative (Tan et al., 2022; Yang et al., 2022), and non-recorded events, which raise data quality issues, should be noted.

By contrast, prospective studies usually provide research ready datasets right from the beginning. These tend to be better curated but also scarce, due to their cost. The number of patients involved can be limited even in multicentric studies; as in Orsini et al. (2017) and Ferrari et al. (2020a), where the few patients were unevenly enrolled across Modena, Hong Kong, and Sydney hospitals. Scarcity and sparsity are not always in contrast, and methods exist to limit both their effects (Bansal et al., 2021).

In clinical datasets, data scarcity/sparsity often conspires with data imbalance. Medical studies often focus on rare or less frequent cases (fewer ill than healthy people, fewer high-than low-risk conditions). For example, in Mandreoli et al. (2021), Hidden Markov Models (HMMs) were used to predict oxygen-therapy state-transitions. Since relatively few patients required *intubation* it was difficult for the Baum et al. (1970) and Welch (2003) algorithm to accurately learn probability distributions. All these problems compromise the learning process of most ML models, including Neural Networks (NNs). Specifically, missing data are not tolerated in the training set, while data scarcity and imbalance lead to overfitting, preventing NNs from generalizing well. Plenty of methods deal with missing data (Penny and Atkinson, 2012), in both descriptive tasks and causal modeling (Sperrin et al., 2020). While missing data can sometimes be inferred/imputed from available feature distributions, this is not an option for critical parameters, like vital ones, that are subject to abrupt changes and thus difficult to predict. Moreover, many popular data imputation methods like MICE (Azur et al., 2011) assume data to be *missing-at-random* to correctly work. This contrasts with the rationale behind “opportunistically” collected datasets, as EHRs, which show patterns of data missingness, as further described in Section 3. This problem raises the need for ML methods tolerant to non-randomly missing data.

Correctly managing data imbalance is a challenge affecting all data science disciplines (Leevy et al., 2018). Although data imputation and augmentation methods can be used to counteract imbalance, it is well-known that they may induce spurious data relations. For example, downsampling the majority class may introduce or remove biases depending on the data quantity/quality as shown by Nwosu et al. (2019). Conversely, upsampling, e.g., using SMOTE by Chawla et al. (2002), is generally acceptable, but excessively amplifying the minority class leads to training sets no longer representing the population ground truth. Other approaches face imbalance by introducing weights into the loss function (Elkan, 2001; Mienye and Sun, 2021). Finally, modifying the study's objectives, following clinical advice, may solve unbalancing problems. This happened in Ferrari et al. (2020c), where next-day respiratory failure risk data were highly imbalanced (30% positives, 70% negatives) whilst next-2-days risk data were not (44% positives, 56% negatives). This clinically-driven adaptation was a non-statistical strategy that effectively prevented an excessive outcome imbalance while also making the outcome clinically safer.

Imbalance and data scarcity may also be tackled together with data augmentation techniques, i.e., combining limited labeled data with synthetic data. While this line of research is still in its infancy, interesting advances concern the development of generative models (Zhang et al., 2017; Jordon et al., 2019). A promising research perspective would be to develop techniques capable of filling gaps in clinical trials, such as missing or underrepresented groups.

### 2.2. (In)stability

In-the-wild data collection routines often change over time, jointly evolving with the clinical practice. Data acquisition and management are affected by emergencies, changes in public policy, hospital resources, and collection technologies. Changes in a tabular dataset schema may cause data instability issues. For instance, in Ferrari et al. (2020c) and Mandreoli et al. (2021), tests were administered daily, depending on the patient's condition and the constantly evolving scientific evidence. The introduction of new biomarkers, like interleukin-6, in patient evaluation came after weeks, causing their absence in the first study dataset.

Algorithm or hyper-parameter fixes momentarily solve instability only when data grows under a constant schema. However, they may not suffice when data collection becomes unstable, eventually changing the model outcomes and interpretation too, propagating instabilities to the deployment stage and thus requiring constant maintenance.

The strategies we identified to address/mitigate data instability are:

1. preventively designing data collection processes and databases to embrace changes from the clinical practice without altering the underlying schema;
2. continuously promoting the cross-talk between physicians and data engineers, to ensure quick answers to the clinical needs without disrupting operational/technological procedures;
3. fortifying debugging facilities, e.g., allowing to capture and query the provenance of the transformations produced by the pipeline.

## 3. Translational challenges

Developing and applying any AI technique in medicine requires the collaboration of physicians and data scientists to produce efficient, robust, and reliable solutions. The former need to understand the assumptions and rationale behind models to provide valuable insights from their domain of expertise; the latter, should learn the clinical significance of data to work on problems of real interest, and build functional and reliable applications. The next subsections, summarize two prominent problems arising from this medicine/AI interface.

### 3.1. Not all errors are equally wrong

High-stakes domains constantly dealing with people's health and safety, like medicine, engineering, or law, must balance any forecast error based on its quality and severity, penalizing the most unwanted or unfair ones; e.g., underestimating risk is much more dangerous than overestimating it. For example, consider binary classification problems where false positive (FP) and false negative (FN) routinely drive predictive performance. When their human and financial cost is balanced, standard measures like AUC-ROC or F-score may correctly measure performance. However, high-risk medical applications often prefer some type of error.

For example, in Ferrari et al. (2020b) the objective was predicting the occurrence of respiratory failure in Covid–19 patients. Tuning the loss function reduced the impact of FN, e.g., patients' predicted to be safe but eventually becoming more severe. Thus, before choosing the final model formulation, it is always necessary to understand the level of risk reduction tolerated by the clinical setting. This issue highlights the need for adequate performance metrics. The AI branch dealing with uneven classification errors and costs is named “cost-sensitive learning” (Turney, 2002; Lomax and Vadera, 2013). There, a cost matrix is defined or learned to reflect the penalty of miss-classifying samples (Wang et al., 2018); other sources of cost/risk can be incorporated in the analysis, as in Freitas et al. (2007). Opposite to re-sampling techniques, cost-sensitive methods can deal with imbalanced datasets without altering the original data distribution (Elkan, 2001; Mienye and Sun, 2021).

Assessing model quality should always come with a study of the errors quality. Indeed, model comparison and evaluation should not be done with just prediction performance in mind, but also considering model fairness (Chouldechova and Roth, 2018; Mehrabi et al., 2021). Unfairness may be induced by biases already present at dataset construction time. Indeed, EHRs often reflect the demographic and ethnic characterization of a country and may therefore contain poorly sampled or totally missing minorities, leading to representation or sampling bias (Suresh and Guttag, 2021). Sensitive attributes like age, sex, ethnicity, or behavioral risk-factors may identify these subgroups. Population bias coupled with data scarcity, generate setups where under/over-sampling techniques usage is challenging. The same holds for down-weighting/discarding sensitive attributes from the analyses (Zeng et al., 2017), or for the extraction of records not containing sensitive information (Zemel et al., 2013; Feldman et al., 2015). Moreover, the aforementioned approaches imply that the sensitive attribute influence on the decision is entirely unfair, although this is not always the case. Indeed, when sensitive data constitute risk-factors for a given disease, their elimination may penalize minorities/subgroups as performance metrics always provide an average value (Suresh and Guttag, 2021).

A trending strategy to mitigate data scarcity and population bias is Federated Machine Learning (FedML). Introduced by Konečnỳ et al. (2016), FedML has proven to be a promising methodology, allowing the distribution of data analysis pipelines across multiple centers without any form of centralization, yet being able to train competitive ML models, able to provide even better performance. Its main advantages are: privacy preservation, since clinical data are not moved, and the possibility of integrating multiple data sources. This increases the dataset diversity, the trained model robustness, and the number of representatives in each group.

Nevertheless, not all population biases can be mitigated through FedML, e.g., people with disabilities will always be a minority. Understanding how and why a sensitive attribute influences other variables in a dataset can be challenging. Recently, Caton and Haas (2020) and Mehrabi et al. (2021) listed various techniques to counteract different sources of bias which make models unfair. Theoretically, algorithms should be able to treat similar individuals similarly and do not discriminate solely based on sensitive data. Checking the algorithm fairness across sub-groups to limit the population bias is essential in medicine. This may be done, e.g., introducing constraints to limit fluctuations in performance on different subgroups, like FP/FN rates (Kearns et al., 2019).

### 3.2. Human in-the-loop AI

Lately, medical and AI progress have had very different speeds. The rapid evolution and diffusion of AI, not always associated with a better understanding of the techniques and their results, highlights how these fields are physiologically different and explains why many resistances from physicians slow down the integration between the two domains. In high-stakes domains, focusing solely on model accuracy without understanding how the result was obtained has already exposed end users to high risks. A series of retrospective analyses highlighting how black-box models led to harmful or unfair decisions can be found in Rudin (2019) (and references therein).

Since clinicians are the end users of DD tools in clinical practice, they must be empowered to interpret predictions correctly to improve their decision making process. However, ML techniques are notoriously difficult to understand and often seen as black-boxes, providing unintelligible answers. The closer predictive models come to being adopted as part of the clinical practice, the more urgent interpretability or, at least, explainability becomes. Recently, much effort was devoted to providing explanations for non-linear models as in Lundberg and Lee (2017) and for Deep Learning models trained on medical images as in Singh et al. (2020). For example, in Ferrari et al. (2020a,c), results were explained using SHapley Additive exPlanations values (SHAPs) by Lundberg et al. (2020), allowing to evaluate feature ranking both at a global and individual level, providing clinical interpretations of the results and highlighting anomalies. Furthermore, since explainability highly depends on the user's background, in Zhang et al. (2020) an empirical framework is produced by quantifying the users' subjective interpretability, taking their feedback into account (e.g., *via* surveys). Nevertheless, although some techniques can explain complex models' results, this is not always sufficient to understand how an outcome is achieved and its real meaning. Several studies show that interpreting models' results is not equivalent to use intrinsically intelligible algorithms (Rudin, 2019). This is why, lately, increasingly more ML works in healthcare started focusing on interpretable ML techniques (Ahmad et al., 2018; Abdullah et al., 2021). Intelligibility was deemed obtainable only at the cost of lowering the model expressive power and thus performances, but recent studies show that this is not the case (Bell et al., 2022).

A model which can “explain itself” only addresses half of the problem. In a true human-in-the-loop AI scenario, physicians' deep understanding of clinical practice should be injected into the ML models, e.g., by expressing agreement/disagreement with the prediction, or a preference for a given error type. Providing these feedback is usually possible, but only at a technical level, at which physicians are not comfortable operating. Flexible graphical interfaces, like those provided by Causal Machine Learning (CML) (Oneto and Chiappa, 2020; Sanchez et al., 2022) could overcome this problem. Determining causal relationships often requires carefully designed experiments since there is a limit to how much can be learned by purely observational data. CML makes it very easy to visualize and reason about complex scenarios. Moreover, it allows wondering, in a *what-if* manner, about potential effects of interventions at population and individual levels. Other interesting research directions bringing humans in-the-loop, concern approaches able to manage structured data and knowledge graphs such as neural symbolic programming and graph neural networks (Lamb et al., 2020).

Currently, iterative processes, where models provide understandable explanations and gradually improve based on user feedback, are not yet routinely applied in data science for healthcare. To lay the foundations for this continuous bidirectional exchange of information, we should now systematize the dialogue between physicians and data scientists. This could soon lead to the assimilation of ML techniques in clinical practice, equating them to the statistical ones, with which they share many theoretical foundations.

## 4. Discussion

In this manuscript, we have summarized the characteristics of health data collected in-the-wild and specifically designed for research purposes. Based on the current literature and our recent experiences in this field, we outlined the main challenges arising when applying DM and ML techniques to healthcare data and the possible methods to solve them.

Firstly, we described issues intrinsic to clinical data usage; these include sparsity, i.e., the lack of data, scarcity, i.e., the limited amount of available data, and imbalance, i.e., the uneven distribution of data samples with regard to outcomes of interest. We also highlighted the impact of data instability, caused by constantly evolving data collection processes in healthcare information systems.

Secondly, we introduced translational challenges, like dealing with the importance of prediction errors and joining the work of clinicians and data scientists in a human-in-the-loop paradigm.

With this paper, we want to promote a more aware approach to clinical data science, where the clinical perspective and DM procedures fit into each other more. Indeed, in our experience, the tightest the interaction between physicians and data scientists, the faster the results will satisfy both the research fields, eventually leading to the discovery of new clinical knowledge and new ML methodologies.

## Data availability statement

The data analyzed in this study is subject to the following licenses/restrictions: datasets from the referred studies (Ferrari et al., 2020a,b,c; Mandreoli et al., 2021), brought as examples from our experience, will not be published since they contain personal information.

## Author contributions

FMa and PM conceptualized and structured this paper. All authors contributed to the drafting, revision of the manuscript, and approved the submitted version.

## Funding

This research was partly funded/supported by the National Institute for Health and Care Research (NIHR) Biomedical Research Centre based at Guy's and St. Thomas' NHS Foundation Trust and King's College London and/or the NIHR Clinical Research Facility.

## Conflict of interest

The authors declare that the research was conducted in the absence of any commercial or financial relationships that could be construed as a potential conflict of interest.

## Publisher's note

All claims expressed in this article are solely those of the authors and do not necessarily represent those of their affiliated organizations, or those of the publisher, the editors and the reviewers. Any product that may be evaluated in this article, or claim that may be made by its manufacturer, is not guaranteed or endorsed by the publisher.

## Author disclaimer

The views expressed are those of the author(s) and not necessarily those of the NHS, the NIHR or the Department of Health and Social Care.
